# Vertical pleiotropy explains the heritability of social science traits

**DOI:** 10.1017/S0140525X22002382

**Published:** 2023-09-11

**Authors:** Charley Xia, W. David Hill

**Affiliations:** Lothian Birth Cohort studies, University of Edinburgh, 7 George Square, Edinburgh EH8 9JZ, UK, Department of Psychology, University of Edinburgh, 7 George Square, Edinburgh, EH8 9JZ, UK; Lothian Birth Cohort studies, University of Edinburgh, 7 George Square, Edinburgh EH8 9JZ, UK, Department of Psychology, University of Edinburgh, 7 George Square, Edinburgh, EH8 9JZ, UK

We contend that social science variables are the product of multiple partly heritable traits. Genetic associations with socio-economic status (SES) may differ across populations, but this is a consequence of the intermediary traits associated with SES differences also varying. Furthermore, genetic data allows the social scientist to make causal statements regarding the aetiology and consequences of SES.

Burt (2022), describes the signal captured by a polygenic score (PGS) derived from a genome-wide association study (GWAS) on social science traits such as education as being “artificial” and a product of social differences rather than genetic processes. As an example of downward causation, Burt (2022) provides the thought experiment posed by Jencks et al. (1972) where, in a hypothetical scenario, red headed individuals are denied access to an education.

We argue that, just as a PGS captures the aggregate effect of each individual single nucleotide polymorphism (SNP) used in its construction, each SNP from a GWAS conducted on education, captures the aggregate effect of each heritable trait associated to differences in education. This process, referred to as vertical pleiotropy (also known a mediator variable) describes incidences where phenotype A (intelligence for example) is associated with phenotype B (education) and so a genetic variant found to be associated with phenotype A will also be associated with phenotype B (Figure 1).

In Burt’s hypothetical example, red hair would emerge as an intermediary phenotype between genetic inheritance and phenotypic consequence but in real data, childhood intelligence (*r_g_* = 0.72, SE = 0.09)(Hill, Davies, Liewald, McIntosh, & Deary, 2016), health (*r_g_* = 0.56, SE = 0.03)(Hill, Marioni, et al., 2019), ADHD (*r_g_* = -0.54, SE = 0.03) (Hill, Marioni, et al., 2019), and neuroticism (*r_g_* = -0.23, SE = 0.02)(Hill et al., 2020) show consistent and substantial genetic correlations with education and give an indication as to what heritable traits may contribute towards educational attainment. In a multivariate analysis examining the traits that contribute towards education in children, Krapohl et al. (2014) found that intelligence, self-efficacy, school environment, home environment, personality, wellbeing, behavioural problems, and health, collectively explained 75% of the heritability of education.

Vertical pleiotropy also illustrates why some PGS are population specific. When applied to education, a PGS would be population specific insofar as the heritable traits underlying educational attainment are not universal. An example of this was provided by Rimfeld et al. (2018) who showed that a PGS predicted 6.1% of education in post-Soviet Estonia compared with 2.1% in Soviet era Estonia. Furthermore, the total heritability of education in post-Soviet Estonia was estimated to be 37% compared to the Soviet era estimate of 17%. Height was used as a control variable and no significant differences between the heritability estimates was found. These differences were attributed to the rise of a more meritocratic society following the fall of the Soviet Union where individual differences in hard work and ability, which are partly genetically mediated, became the traits predictive of educational success rather than environmentally driven privilege or discrimination.

Some of the heritable traits underlying differences in education may indeed be population specific, as indicated by population specific genetic effects on education (Rimfeld et al., 2018; Tropf et al., 2017). However, meta-analyses of GWAS of education do facilitate loci discovery, which is indicative that some of the association signal is replicated across samples and is consistent with the idea that similar heritable traits underlie education differences across, predominantly European, countries and cultures.

Finally, Burt (2022) asks what the added value is of including genetics in a social science study. Mendelian Randomisation (MR) is a technique that, at its heart, uses vertical pleiotropy to examine if two traits (such as health and education for example) are causally connected. This is achieved by using genetic variants (such as single or multiple SNPs from a GWAS) as instrumental variables for risk factors that affect the health of a population. As genetic variants are fixed at conception their use as instrumental variables can overcome some types of confounding.

Applied to social science variables, MR has helped to understand the causes and consequences of SES differences where intelligence has been putatively shown to be a causal factor for income (Hill, Davies, et al., 2019) and education (Anderson et al., 2020; Davies et al., 2019), where bi-directional casual effects exist the case of education. When applied in a multivariable analysis, MR has indicated that education, and not the highly correlated trait of intelligence is a causal factor in smoking (Sanderson, Davey Smith, Bowden, & Munafò, 2019). Conversely, higher intelligence, and not education, has been indicated to be a causal protective factor against Alzheimer’s disease (Anderson et al., 2020). Using a within-family design an increase in BMI was identified as causally associated to lower levels of education (Howe et al., 2022).

In conclusion, PGS and GWAS conducted on social science traits capture the partly heritable traits that likely contribute to some of the variance of SES. Such associations are as authentic as those that act in biological pathways influencing disease traits, the difference being that, for social science traits, SNP associations are at the start of a phenotypic pathway beginning at molecular genetic inheritance and ending at phenotypic consequence. This pathway can differ between populations, but it is a strength of the molecular genetic design that MR can be applied to examine which heritable traits are causally linked to SES differences across and between cultures.

## Figures and Tables

**Figure 1:**
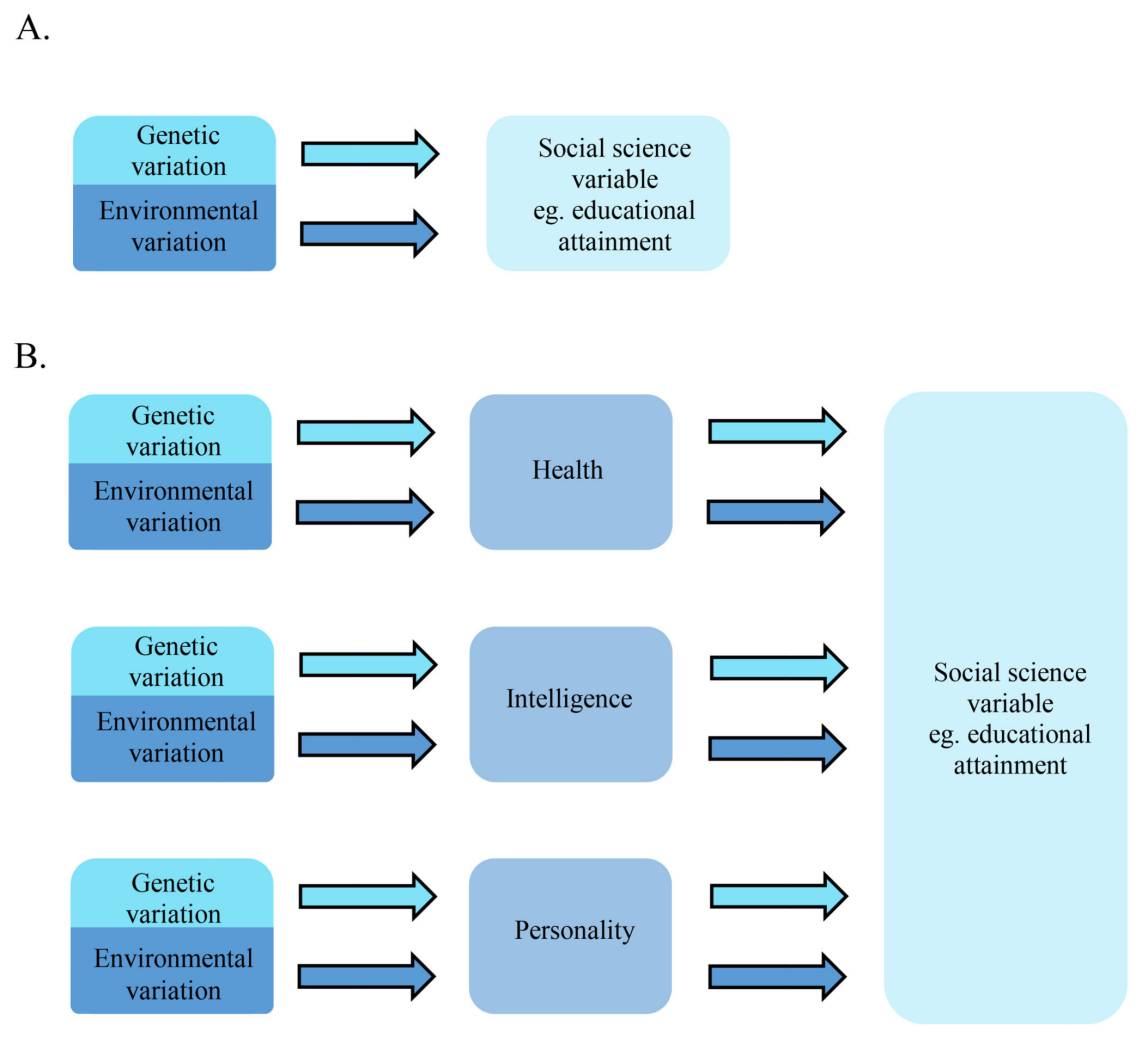
A simplified illustration of vertical pleiotropy showing a subset of the possible intermediary phenotypes between genetic variation and phenotypic differences in social science variables. Illustrated is a schematic describing that when a GWAS is performed on, or a polygenic score is derived to predict differences in, education, genetic variation is linked to education (Panel A.). However, the means by which an association occurs is that, in part, a number of partly heritable traits are themselves associated with education as part of a phenotype pathway starting with genetic inheritance and ending with phenotypic consequences for education (Panel B.). Light blue boxes indicate sources of genetic variation where light blue arrows show the association between genetic and trait variation measured using GWAS or PGS. Dark blue shows sources of environmental variation with dark blue arrows indicating environmental associations with a trait. Pale blue indicates education as an example of a social science variable. The blue/grey boxes in panel B. show possible intermediary heritable phenotypes.

## References

[R1] Anderson EL, Howe LD, Wade KH, Ben-Shlomo Y, Hill WD, Deary IJ, Hemani G (2020). Education, intelligence and Alzheimer’s disease: Evidence from a multivariable two-sample Mendelian randomization study. International Journal of Epidemiology.

[R2] Burt CH (2022). Challenging the Utility of Polygenic Scores for Social Science: Environmental Confounding, Downward Causation, and Unknown Biology. Behavioral and Brain Sciences.

[R3] Davies NM, Hill WD, Anderson EL, Sanderson E, Deary IJ, Davey Smith G (2019). Multivariable two-sample Mendelian randomization estimates of the effects of intelligence and education on health. eLife.

[R4] Hill WD, Davies G, Liewald DC, McIntosh AM, Deary IJ (2016). Age-Dependent Pleiotropy Between General Cognitive Function and Major Psychiatric Disorders. Biological psychiatry.

[R5] Hill WD, Davies NM, Ritchie SJ, Skene NG, Bryois J, Bell S, Deary IJ (2019). Genome-wide analysis identifies molecular systems and 149 genetic loci associated with income. Nature communications.

[R6] Hill WD, Marioni RE, Maghzian O, Ritchie SJ, Hagenaars SP, McIntosh AM, Deary IJ (2019). A combined analysis of genetically correlated traits identifies 187 loci and a role for neurogenesis and myelination in intelligence. Molecular psychiatry.

[R7] Hill WD, Weiss A, Liewald DC, Davies G, Porteous DJ, Hayward C, Deary IJ (2020). Genetic contributions to two special factors of neuroticism are associated with affluence, higher intelligence, better health, and longer life. Molecular psychiatry.

[R8] Howe LJ, Nivard MG, Morris TT, Hansen AF, Rasheed H, Cho Y, Within Family, C (2022). Within-sibship genome-wide association analyses decrease bias in estimates of direct genetic effects. Nature genetics.

[R9] Jencks C, Smith M, Henry A, Jo Bane M, Cohen D, Gintis H, Michelson S (1972). Inequality: A reassessment of the effect of family and schooling in America.

[R10] Krapohl E, Rimfeld K, Shakeshaft NG, Trzaskowski M, McMillan A, Pingault J-B, Plomin R (2014). The high heritability of educational achievement reflects many genetically influenced traits, not just intelligence. Proceedings of the National Academy of Sciences.

[R11] Rimfeld K, Krapohl E, Trzaskowski M, Coleman JRI, Selzam S, Dale PS, Plomin R (2018). Genetic influence on social outcomes during and after the Soviet era in Estonia. Nature Human Behaviour.

[R12] Sanderson E, Davey Smith G, Bowden J, Munafò MR (2019). Mendelian randomisation analysis of the effect of educational attainment and cognitive ability on smoking behaviour. Nature communications.

[R13] Tropf FC, Lee SH, Verweij RM, Stulp G, van der Most PJ, de Vlaming R, Mills MC (2017). Hidden heritability due to heterogeneity across seven populations. Nature Human Behaviour.

